# Epidemiological investigation of porcine *Mycoplasma hyopneumoniae* in pig herds in Guangxi, China (2022–2023) and genetic diversity analysis based on multilocus sequence typing

**DOI:** 10.3389/fvets.2025.1619301

**Published:** 2025-08-13

**Authors:** Ying Zhou, Shuo Zhao, Longhua Liang, Yibin Qin, Ke Cheng, Zhongwei Chen, Bingxia Lu, Qunpeng Duan, Ying Peng, Yu Huang, Zhenhua Duan, Kaiyi Jiang, Xunye Yang, Ying He

**Affiliations:** ^1^Guangxi Key Laboratory of Veterinary Biotechnology, Key Laboratory of China (Guangxi)-ASEAN Cross-Border Animal Disease Prevention and Control, Guangxi Veterinary Research Institute, Ministry of Agriculture and Rural Affairs of China, Nanning, China; ^2^College of Animal Science and Technology, Guangxi University, Nanning, China; ^3^Guangxi National Farm Yongxin Animal Husbandry Group Co., Ltd., Nanning, China; ^4^Yulin Animal Disease Prevention and Control Center, Yulin, China

**Keywords:** *Mycoplasma hyopneumoniae*, genotyping, MLST, swine epidemiology, Guangxi

## Abstract

**Introduction:**

Mycoplasma hyopneumoniae (Mhp) infection significantly challenges Guangxi’s pig farms, yet its prevalence and molecular characteristics remain poorly understood. This study aimed to define circulating Mhp genotypes and their distribution in the region.

**Methods:**

From 2022–2023, 1,362 pig lung samples were randomly collected from 14 Guangxi regions. Mhp was detected using TaqMan Real-time PCR. Strong positive samples underwent multilocus sequence typing (MLST) of adk, rpoB, and tpiA genes to assess genetic relationships.

**Results:**

Of 1,362 samples, 655 (48.1%) were Mhp-positive. MLST amplification succeeded for 61 samples, revealing 27 sequence types (24 novel) across all 14 regions. Phylogenetic analysis indicated predominant circulation of Mhp types I and V. High Mhp incidence and substantial genetic diversity were observed.

**Discussion:**

This study provides comprehensive analysis of Mhp in Guangxi, revealing high prevalence and genetic diversity dominated by types I and V. These findings expand understanding of Mhp epidemiology in China and offer a theoretical foundation for developing prevention and control strategies in Guangxi.

## Introduction

*Mycoplasma hyopneumoniae* (Mhp) is the etiological agent of *Mycoplasma pneumonia* (MPS), also called enzootic pneumonia (EP) in swine, commonly referred to as porcine “wheezing disease” (1). This disease exhibits high morbidity but low mortality, and can appear year-round. Clinical signs primarily include dyspnea, abdominal breathing, lethargy, and coughing, along with decreased growth performance and increased feed conversion ratio (2, 3). Mhp can also act synergistically with other pathogens (4, 5), such as porcine circovirus type 2 and porcine reproductive and respiratory syndrome virus, to produce porcine respiratory disease complex (PRDC) (6, 7), thereby complicating prevention and control efforts, and presenting a challenge to the worldwide swine industry. Guangxi Province, a critical region for swine production in China, faces challenges due to *Mycoplasma hyopneumoniae* (Mhp) infections across its diverse pig farms. Zhang et al. identified Guangxi as a high-prevalence area for Mhp in China during a 2019 outbreak, reporting substantial genetic diversity, including eight novel ST types. Li (8) reported widespread Mhp infection across Chinese pig farms, with a national antigen-positive rate of 19.19%. Guangxi contributed the largest sample size, exhibiting an infection rate comparable to the national average. However, a comprehensive understanding of the prevalence and molecular characteristics of these infections throughout the region remains elusive. Multilocus sequence typing (MLST) has emerged as the gold standard for high-resolution molecular epidemiology of Mhp, enabling robust strain differentiation, phylogenetic analysis, and inter-study comparisons via global databases (9, 10). This method defines stable sequence types (STs) based on nucleotide variations in core housekeeping genes. The analysis of the adk, rpoB, and tpiA genes specifically provides discriminatory power equivalent to the original 7-gene scheme for Mhp (11), making it highly practical for large-scale surveillance and genotyping directly from clinical samples.

This study randomly sampled 1,362 pig lung tissue from 14 cities in Guangxi for pathogen detection. The targeted gene sequences obtained by sequencing were connected in series according to the order of adk-rpoB-tpiA. Genetic evolution tree was drawn for genetic polymorphism analysis to explain the genotype and distribution of Mhp in Guangxi pig herds.

## Materials and methods

### Samples

Between 2022 and 2023, 1,362 lung samples (one per pig) were collected opportunistically from swine at various abattoirs across 14 municipalities in Guangxi Province, negating the need for ethical approval. In order to ensure the scientificity and fairness of sampling and minimize selection bias, we carried out meticulous and rigorous sampling work in 14 cities in Guangxi Province according to multi-dimensional factors such as geographical distribution characteristics, breeding scale and production capacity. In the process of pig farm selection, we comprehensively referred to the breeding record information provided by the agricultural departments of various cities and divided all farms into three categories: large, medium and small according to the breeding scale. Then, a stratified sampling method is used to randomly select a certain number of farms from each level according to an equal proportion, so as to ensure that farms of different sizes have the opportunity to be included in the sample category.

At the same time, the geographical span of each city is fully considered to ensure that the selected farms cover all major breeding clusters to avoid bias caused by geographical concentration. Specifically, samples originated from the following locations: Nanning (*n* = 282), Liuzhou (*n* = 42), Guilin (*n* = 81), Wuzhou (*n* = 110), Beihai (*n* = 76), Fangchenggang (*n* = 80), Qinzhou (*n* = 49), Guigang (*n* = 99), Yulin (*n* = 97), Baise (*n* = 98), Hezhou (*n* = 15), Hechi (*n* = 100), Laibin (*n* = 168), and Chongzuo (*n* = 65).

### DNA extraction, RT-qPCR detection

Lung tissue (0.5 g) was collected in a 2 mL enzyme-free Eppendorf tube and minced. Four grinding beads and approximately 1 mL of physiological saline were added to the tube. Each sample was homogenized twice at 70 Hz for 70 s utilizing a tissue homogenizer. DNA from Mhp was extracted from the homogenates utilizing an Automatic Nucleic Acid Extraction Machine (Zybio Technology Company, Chongqing Province, China). Mhp was detected utilizing a highly specific and sensitive qPCR assay (12). Primers and probe sequences were as follows: Mhp 183-F: CAAAGCGAGTATGAAGAACAAGAAA; Mhp 183-R: GTCATCATTGGGTGGCTAAGT; Mhp183-ROX-TCCAGGAAGTCAAGGTAACTAGTGACCA-BHQ. qPCR reactions were carried out in a 20 μL reaction volume consisting of 10 μL 2× Taqman Fast qPCR Mastermix (Sangong, Shanghai, China), 0.4 μL each of primers Mhp 183-F and Mhp 183-R, 0.3 μL of probe Mhp 183-P, 4 μL of cDNA, and 4.8 μL of nuclease-free water. Samples exhibiting strong positive results (Ct-value <30) were eligible for direct genotyping (13).

The reaction system is as follows:

**Table:** 

Name	Volume
2× TaqMan Fast qPCR Master mix	10
Mhp183-F	0.4
Mhp183-R	0.4
Mhp183-P	0.3
cDNA template	2
Nuclease-free Water	6.9

The reaction system is as follows:

**Table:** 

Procedure	Temperature (°C)	Time (s)	
Predegeneration	94	120	
Degeneration	94	30	$\}30\text{cycles}$
Anneal	57	30	
Extend	72	60	
Terminal extension	72	420	
Predegeneration	94	120	

### MLST

Nucleotide sequence data for the adk, ropB, and tpiA genes were generated from Mhp-positive samples through Sanger sequencing. These genes were amplified utilizing primers and conditions previously described by Mayor et al. Amplicons were resolved by electrophoresis on a 1.5% agarose gel and purified utilizing the SanPrep Column DNA Gel Extraction Kit (Sangon, Shanghai, China).

Sequencing was performed by Sangon Biotech (Shanghai, China). The resulting sequence data were deposited in the PubMLST database for sequence type (ST) assignment. Each housekeeping gene receives a unique allele number, and the combination of the three alleles determines a specific ST.

### Guangxi, China

Guangxi Province is located in southern China, bordered by Yunnan to the west, Guizhou to the north, Hunan to the northeast, and Guangdong to the southeast. Its topography comprises a diverse range of landscapes, from mountains and karst formations to rivers and fertile plains. As presented in Supplementary Figure 1, the northern region of Guangxi (Baise, Hechi, Liuzhou, and Guilin) is primarily mountainous. Guangxi plays a crucial role in China’s pork production; the region has a large agricultural sector, with pig farming as a major component. In effect, Guangxi ranks as a leading province for pig farming and is a significant contributor to the national pork supply.

### Data analysis

We obtained the allele data for adk, rpoB, tpiA and ST from the PubMLST database.^1^ We calculated Simpson’s index of diversity values utilizing the website.^2^ We carried out sequence alignment and cluster analysis through Molecular Evolutionary Genetics Analysis (MEGA version 6.0) utilizing the neighbour-joining method (Kimura 2-parameter model).

## Results

### Survey on Mhp positivity rate in Guangxi

**Figure 1:**
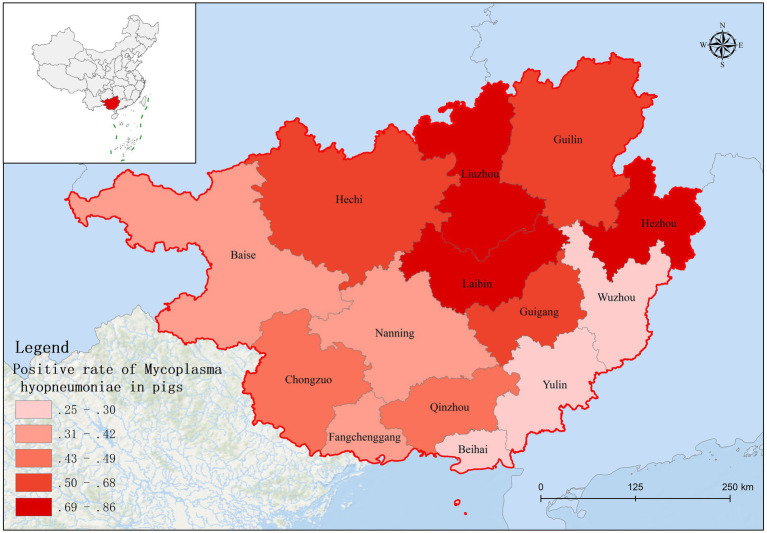
The distribution of Mhp positive rate in Guangxi Province.

**Figure 2:**
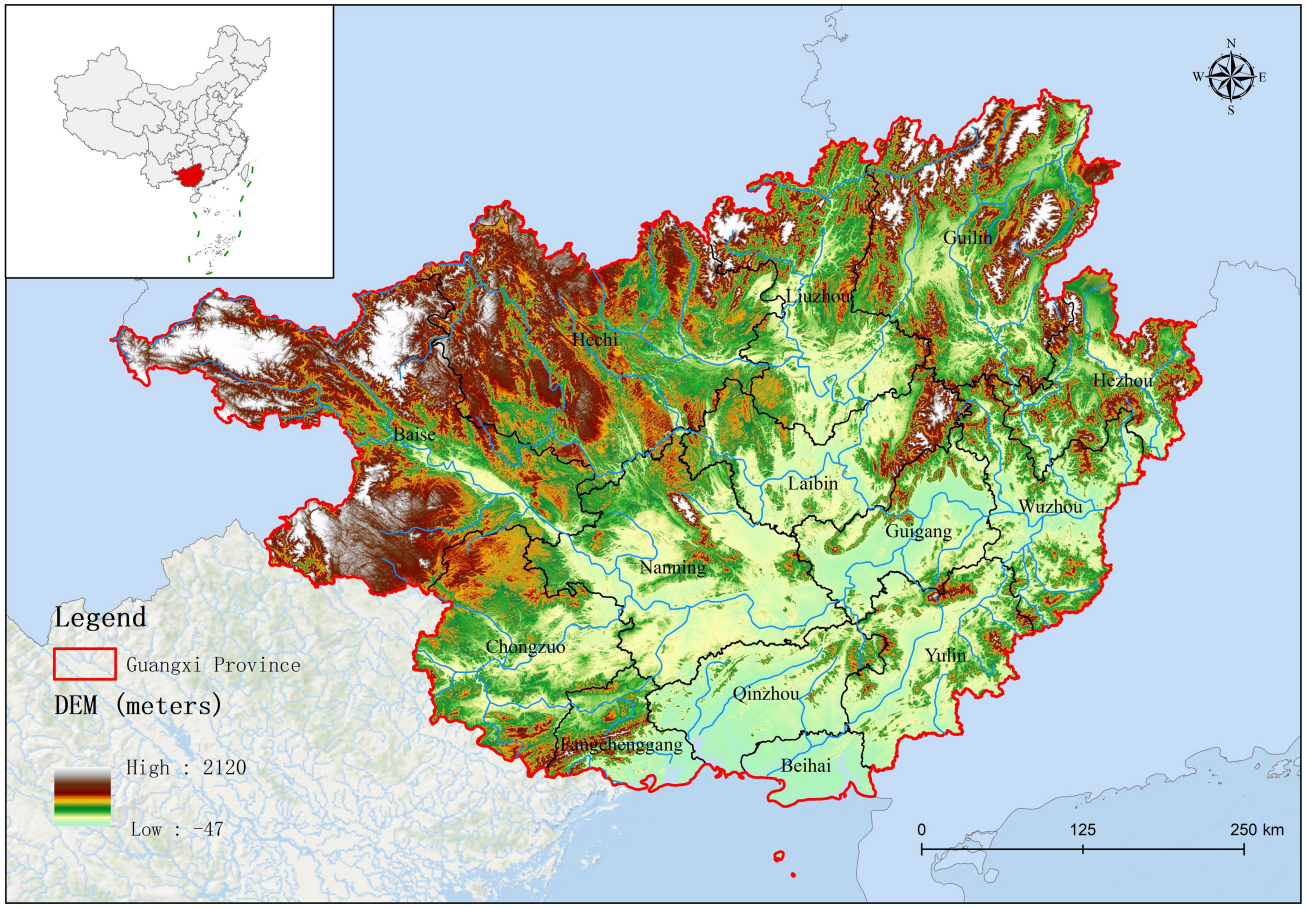
The topographical map of Guangxi Province.

Based on fluorescence quantitative PCR results, we identified 655 Mhp-positive samples out of 1,362 lung tissue samples collected from 14 municipalities in Guangxi. Six regions demonstrated notably high positive rates: Liuzhou 85.71% (36/42), Hezhou 80.00% (12/15), Laibin 85.12% (143/168), Hechi 68.00% (68/100), Guigang 64.65% (64/99), and Guilin 58.02% (47/81). These six regions all maintained positivity rates above 50%. Across the entire Guangxi region, the overall Mhp positivity rate reached 48.09% (655/1,362). We discovered three regions with low Mhp positivity rates: Yulin 29.90% (29/97), Wuzhou 28.18% (31/110), and Beihai 25.00% (19/76), with all these regions indicating rates below 30% (Table 1). Our spatial analysis indicated that in the northern part of the Guangxi region, Laibin, Liuzhou, Hezhou, Guilin, and Hechi displayed higher Mhp positivity rates. In contrast, the southeastern areas of Wuzhou, Yulin, and Beihai, which lie next to Guangzhou, demonstrated relatively lower Mhp positivity rates (Figure 1). Our terrain analysis demonstrated that the northern regions of Guangxi, which had the previously mentioned higher Mhp-positive rates, featured higher terrain and primarily mountainous landscapes (Figure 2).

**Table 1:** Positive detection of Mhp in various cities in Guangxi.

Regions	Positive count of Mhp	Positivity rate of Mhp
Liuzhou	36/42	85.71%
Laibin	143/168	85.12%
Hezhou	12/15	80.00%
Hechi	68/100	68.00%
Guigang	64/99	64.65%
Guilin	47/81	58.02%
Chongzuo	32/65	49.23%
Qinzhou	22/49	44.90%
Baise	41/98	41.84%
Nanning	114/282	40.43%
Fangchenggang	28/80	35.00%
Yulin	29/97	29.90%
Wuzhou	31/110	28.18%
Beihai	19/76	25.00%
Guangxi	655/1362	48.09%

**Figure 3:**
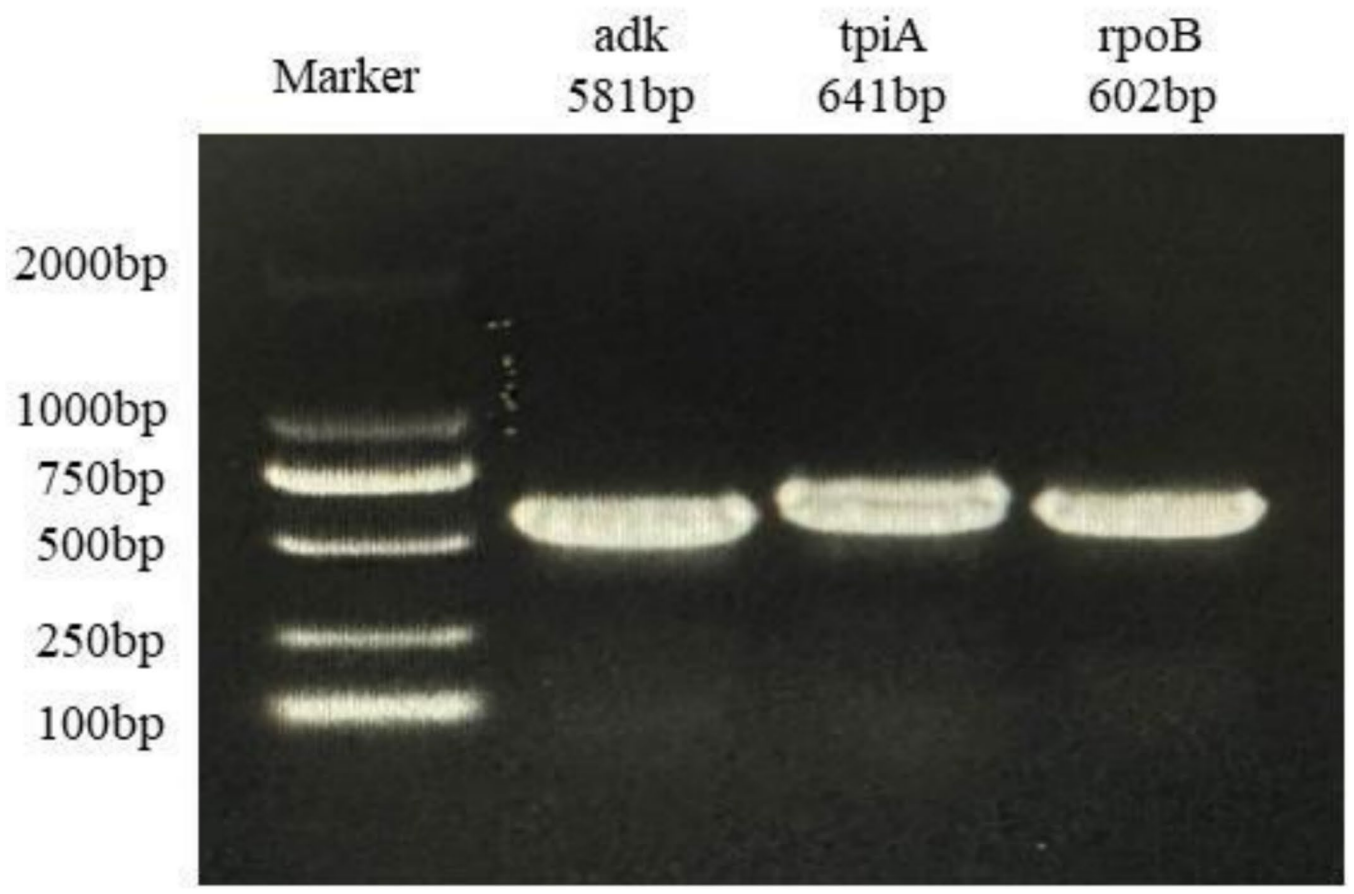
Agarosegel electrophoresis of PCR amplification of Mhp housekeeping gene adk, tpiA, and rpoB.

**Figure 4:**
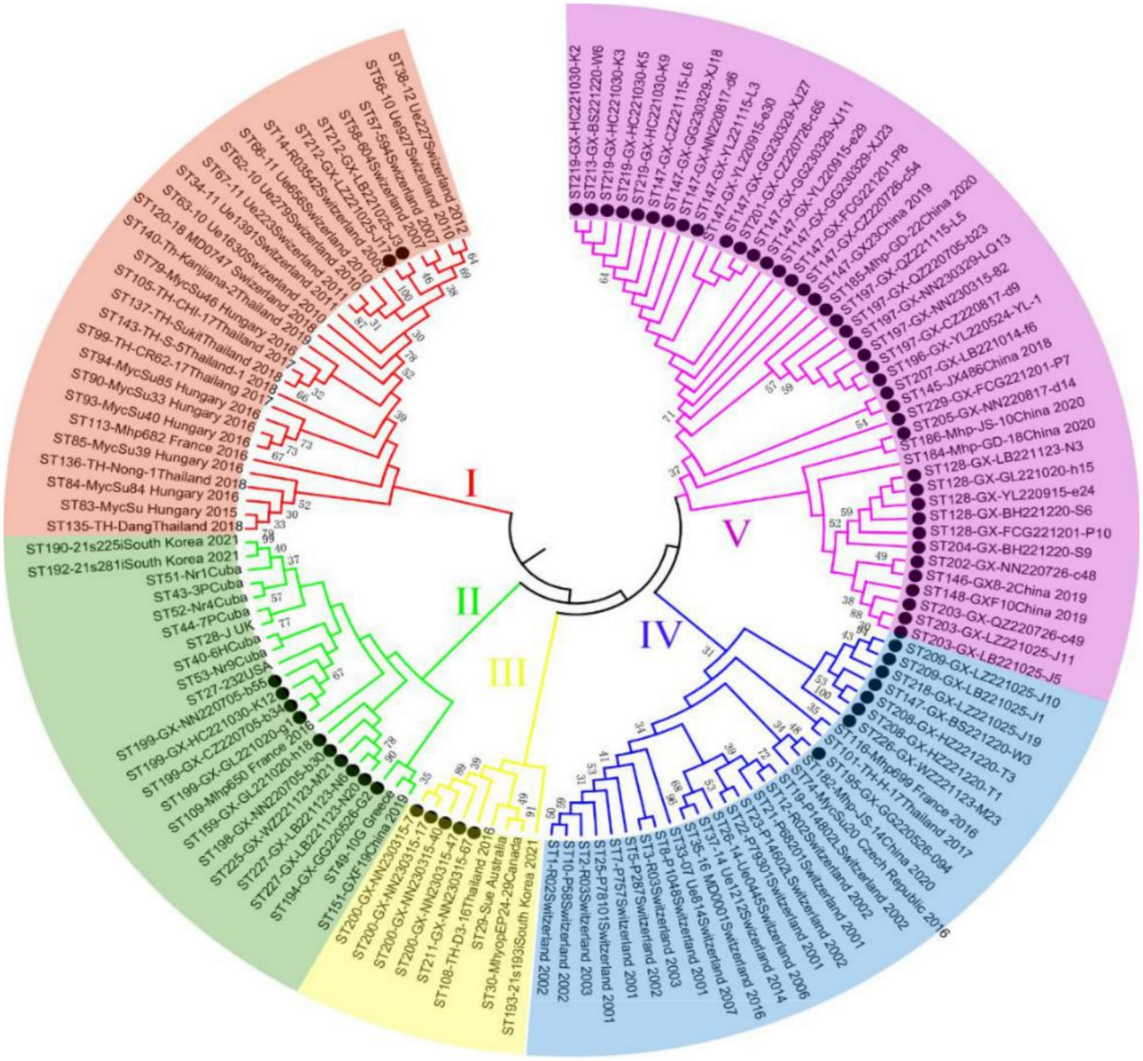
The neighbor-joining tree of selected sequence types (STs) based on the adk, rpoB, and tpiA sequences of Mhp in Guangxi. ● Represents the isolates in this experiment.

**Figure 5:**
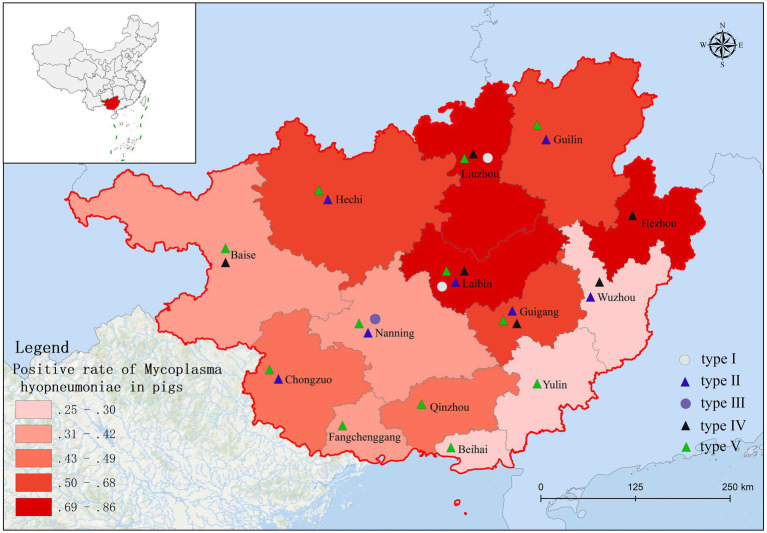
Distribution of the five genotypes of Mhp in Guangxi Province.

Of 655 PCR-positive samples (Ct <30) subjected to amplification of key MLST genes (adk, rpoB, tpiA), only 61 (9.3%) were successful, yielding a 90.7% failure rate. Figure 3 presents the gel electropherograms depicting the simultaneous amplification of these three housekeeping genes. Analysis of the alleles from the successfully amplified samples indicated the identification of two novel *adk* alleles (56, 57), 10 novel *rpoB* alleles (72, 73, 74, 75, 76, 77, 79, 80, 81, 82), and three novel *tpiA* alleles (82, 83, 84). These 61 samples were grouped into 27 ST types based on the allelic profiles of *adk*, *rpoB*, and *tpiA* (128, 147, 159, 194, 195, 196, 197, 198, 199, 200, 201, 202, 203, 204, 205, 207, 208, 209, 211, 212, 213, 218, 219, 225, 226, 227, 229). Of these STs, 24 are newly described. Specifically, ST147 and ST128 were prevalent, observed in six geographically proximate cities (Baise, Chongzuo, Nanning, Fangchenggang, Yulin, and Guigang) and five more geographically dispersed cities (Nanning, Guilin, Laibin, Fangchenggang, Beihai, and Yulin), respectively. In addition, ST197, ST199, and ST203 were found in multiple regions. The Simpson index of diversity for STs is 0.939 (Table 2). Phylogenetic analysis of ST sequences resolved the 61 strains into five genotypes (types I, II, III, IV, and V). Type I comprised strains from Switzerland, Hungary, and Thailand, as well as two strainsfrom this study. Type II comprised 10 strains from this study, representing seven regions of Guangxi: Nanning, Hechi, Chongzuo, Guilin, Wuzhou, Laibin, and Guigang. This distribution suggests that type Mhp is prevalent in half of Guangxi. In addition, a phylogenetic relationship was observed between the UK strain J, the US strain 232, and the following strains from this study: ST194, ST227, ST225, ST198, ST199, and ST159. Type III was found only in the Nanning area, along with strains from Thailand, Canada, Australia, and South Korea. Type IV consisted of eight strains from this study, detected in Liuzhou, Laibin, Guigang, Wuzhou, Hezhou, and Baise. These strains demonstrated a closer evolutionary relationship with a 2016 French strain, a 2017 Thai strain, and a 2020 Jiangsu strain than with the Swiss strains belonging to a different genotype. Type V, the largest genotype in this study, was identified in nearly every region of Guangxi. This genotype also included a 2018 Jiangxi strain (JX486), 2019 Guangxi strains (GX23, GX8-2, and GXF10), 2020 Guangdong strains (GD-22, GD-18), and a 2020 Jiangsu strain (JS-10) (Figure 4). Geospatial analysis demonstrated a limited distribution of type I, exclusively in Laibin and Liuzhou, whereas type II exhibited a broader presence across Guangxi, represented by an “x” shaped distribution across the northeastern and southwestern regions. The four type III strains originated from a single farm located in Nanning. Type IV was primarily located in the northeastern quadrant of Guangxi, including Liuzhou, Hezhou, Wuzhou, Laibin, and Guigang. Type V was observed in all areas with the exception of Wuzhou and Hezhou (Figure 5).

**Table 2:** The summary of positive herds by date and genotyping result.

Herd ID	Country and region of herd	Year	adk	rpoB	tpiA	ST
GX-GL221020-h15	Guilin, Guangxi, China	2022	23	59	57	128
GX-LB221123-N3	Laibin, Guangxi, China	2022	23	59	57	128
GX-FCG221201-P10	Fangchenggang, Guangxi, China	2022	23	59	57	128
GX-BH221220-S6	Beihai, Guangxi, China	2022	23	59	57	128
GX-YL220915-e24	Yulin, Guangxi, China	2022	23	59	57	128
GX-CZ220726-c54	Chongzuo, Guangxi, China	2022	16	15	59	147
GX-NN220817-d6	Nanning, Guangxi, China	2022	16	15	59	147
GX-YL221115-L3	Yulin, Guangxi, China	2022	16	15	59	147
GX-CZ221115-L6	Chongzuo, Guangxi, China	2022	16	15	59	147
GX-FCG221201-P8	Fangchenggang, Guangxi, China	2022	16	15	59	147
GX-BS221220-W3	Baise, Guangxi, China	2022	16	15	59	147
GX-YL220915-e29	Yulin, Guangxi, China	2022	16	15	59	147
GX-YL220915-e30	Yulin, Guangxi, China	2022	16	15	59	147
GX-GG230329-XJ11	Guigang, Guangxi, China	2023	16	15	59	147
GX-GG230329-XJ18	Guigang, Guangxi, China	2023	16	15	59	147
GX-GG230329-XJ23	Guigang, Guangxi, China	2023	16	15	59	147
GX-GG230329-XJ27	Guigang, Guangxi, China	2023	16	15	59	147
GX-GL221020-h18	Guilin, Guangxi, China	2022	41	15	58	159
GX-GG220526-G2	Guigang, Guangxi, China	2022	56	15	50	194
GX-GG220526-094	Guigang, Guangxi, China	2022	20	72	58	195
GX-YL220524-YL-1	Yulin, Guangxi, China	2022	23	73	59	196
GX-QZ220705-b23	Qinzhou, Guangxi, China	2022	23	15	59	197
GX-CZ220817-d9	Chongzuo, Guangxi, China	2022	23	15	59	197
GX-QZ221115-L5	Qinzhou, Guangxi, China	2022	23	15	59	197
GX-NN230315-82	Nanning, Guangxi, China	2023	23	15	59	197
GX-NN230329-LQ13	Nanning, Guangxi, China	2023	23	15	59	197
GX-NN220705-b30	Nanning, Guangxi, China	2022	16	59	58	198
GX-CZ220705-b34	Chongzuo, Guangxi, China	2022	16	74	15	199
GX-NN220705-b55	Nanning, Guangxi, China	2022	16	74	15	199
GX-GL221020-g1	Guilin, Guangxi, China	2022	16	74	15	199
GX-HC221030-K12	Hechi, Guangxi, China	2022	16	74	15	199
GX-NN230315-7	Nanning, Guangxi, China	2023	16	15	84	200
GX-NN230315-17	Nanning, Guangxi, China	2023	16	15	84	200
GX-NN230315-40	Nanning, Guangxi, China	2023	16	15	84	200
GX-NN230315-47	Nanning, Guangxi, China	2023	16	15	84	200
GX-CZ220726-c65	Chongzuo, Guangxi, China	2022	16	75	59	201
GX-NN220726-c48	Nanning, Guangxi, China	2022	16	76	57	202
GX-QZ220726-c49	Qinzhou, Guangxi, China	2022	41	77	57	203
GX-LB221025-J5	Laibin, Guangxi, China	2022	41	77	57	203
GX-LZ221025-J11	Liuzhou, Guangxi, China	2022	41	77	57	203
GX-BH221220-S9	Beihai, Guangxi, China	2022	23	23	57	204
GX-NN220817-d14	Nanning, Guangxi, China	2022	16	79	57	205
GX-LB221014-f6	Laibin, Guangxi, China	2022	41	54	83	207
GX-HZ221220-T1	Hezhou, Guangxi, China	2022	16	81	76	208
GX-HZ221220-T3	Hezhou, Guangxi, China	2022	16	81	76	208
GX-LB221025-J1	Laibin, Guangxi, China	2022	16	58	59	209
GX-LZ221025-J10	Liuzhou, Guangxi, China	2022	16	58	59	209
GX-NN230315-67	Nanning, Guangxi, China	2023	23	59	84	211
GX-LB221025-J3	Laibin, Guangxi, China	2022	16	58	82	212
GX-LZ221025-J17	Liuzhou, Guangxi, China	2022	16	58	82	212
GX-BS221220-W6	Baise, Guangxi, China	2022	57	82	59	213
GX-LZ221025-J19	Liuzhou, Guangxi, China	2022	16	80	59	218
GX-HC221030-K2	Hechi, Guangxi, China	2022	57	15	59	219
GX-HC221030-K3	Hechi, Guangxi, China	2022	57	15	59	219
GX-HC221030-K5	Hechi, Guangxi, China	2022	57	15	59	219
GX-HC221030-K9	Hechi, Guangxi, China	2022	57	15	59	219
GX-WZ221123-M21	Wuzhou, Guangxi, China	2022	38	59	58	225
GX-WZ221123-M23	Wuzhou, Guangxi, China	2022	16	18	57	226
GX-LB221123-N6	Laibin, Guangxi, China	2022	38	15	58	227
GX-LB221123-N20	Laibin, Guangxi, China	2022	38	15	58	227
GX-FCG221201-P7	Fangchenggang, Guangxi, China	2022	16	59	59	229
Simpson index			0.656 (CI: 0.549–0.763)	0.750 (CI: 0.644–0.855)	0.739 (CI: 0.648–0.831)	0.939 (CI: 0.907–0.972)

## Discussion

A total of 1,362 samples were randomly collected from 14 different regions throughout Guangxi Province. Analysis by real-time fluorescence quantitative PCR identified 655 samples as positive for *Mycoplasma hyopneumoniae* (Mhp). This finding clearly demonstrates a serious Mhp infection problem in Guangxi. In particular, the positive rates in Liuzhou, Laibin, Hezhou, Hechi, Guigang, and Guilin exceeded 50%, indicating that these areas are the most severely affected by Mhp infection in Guangxi. The relatively high positive rates in Liuzhou, Laibin, Hezhou, Hechi, and Guilin may be closely related to their geographical characteristics. Compared to southern Guangxi, the northern part of the province has a higher latitude, lower temperatures, more mountainous terrain, and greater humidity. Previous work by Goodwin (14) and Browne et al. (15) has presented that Mhp is more likely topersist in environments with low temperatures and high humidity (16). Studies have demonstrated a strong association between the prevalence of this pathogen and seasonal variations, as well as a significant effect of climate factors (17, 18). For instance, research conducted in Belgium and the Netherlands found that the infection rate of *Mycoplasma hyopneumoniae* in swine differed seasonally, with a significantly higher infection rate in pigs born in autumn (19). Moreover, environmental factors, including temperature and humidity, greatly affect disease transmission. The spread of the pathogen may be facilitated by the creation of bioaerosols (20), notably during cold and wet seasons (18, 21). While vaccination has significantly offered some protection to pigs against Mhp, these protective effects are known to differ significantly across pig herds. This variability may arise from a range of factors, including the Mhp infection level in the herd, the age of infection onset in pigs, and even potential variations among Mhp isolates (22, 23). Notwithstanding such variability, vaccination is still broadly considered the most effective method for managing Mhp infection (24). These vaccines primarily reduce clinical signs by stimulating an immune response; however, they cannot entirely prevent infection or viral shedding (25, 26). It is important to understand that, while vaccination can successfully reduce clinical signs and lung lesions in infected pigs, its ability to limit the transmission of Mhp is quite restricted (22, 27). This limited capacity to control Mhp spread may be a key factor contributing to the challenges in effectively managing and controlling Mhp. In addition, a positive Mhp test result currently does not restrict the commercial trade of live pigs. Guangxi is a major pig-producing province in China, and live pig trade extends to adjacent provinces such as Guangdong and Jiangxi. This expansive trade network may contribute to the spread of Mhp across different regions. Pig trade is also an important route for the spread of pathogens between countries, which is common on many continents (28). Currently, Mhp is widespread in Guangxi Province. Specifically, Mhp positive rates in Guigang, Chongzuo, and Nanning are comparatively high. This might be due to that these areas are major pig production hubs in Guangxi. These combined factors likely contribute to the high Mhp infection rate and genetic diversity in the province. In this research, we performed a detailed genotyping analysis analyzing Mhp strains located in Guangxi, China. In our collection of 655 positive samples, we observed that 61 samples demonstrated concurrent amplification patterns of the adk, rpoB, and tpiA genes. When conducting multilocus sequence typing (MLST) assays of Mhp, the standard protocol traditionally requires that seven housekeeping genes must be amplified in a parallel fashion. Nevertheless, extensive research has consistently demonstrated that the discriminatory capabilities of the three genes mentioned above match those achieved with all seven housekeeping genes (29–31). For an overwhelming number of samples in this study, we regularly encountered situations where, likely due to insufficient nucleic acid content of Mhp present in the tissue samples or because of various PCR inhibitors in the samples (9, 32), we could not achieve successful simultaneous amplification and sequencing of all seven housekeeping genes. Our analysis indicated new sequence type (ST) distribution patterns throughout the 14 regions that constitute Guangxi Province. We discovered that particular ST classifications, specifically ST197, ST199, and ST203, were located across multiple separate regions. Across Guangxi Province, a considerable distance of approximately 520 kilometers separates Guilin (in the northeast) and Chongzuo (in the southwest). This geographic distribution suggests that distance alone does not significantly impede Mhp transmission.

Infected and asymptomatically infected pigs are the primary infection reservoirs, with sows and latently infected pigs representing the key vectors in farms. Mhp spreads horizontally across long distances through direct pig contact, or through contaminated excretions, oral secretions, and even aerosols (33, 34). Therefore, the inter-regional movement of pigs likely contributes to Mhp dissemination. Zhang et al. (35) first identified ST147 in Guangxi in 2019. However, another study indicated that ST128 was the major ST circulating in Guangxi during that period. By early 2022, Yiming (36) confirmed ST128 as the epidemic strain across seven infected pig farms in Guangxi. This study also identified ST197, ST199, and ST203 in various locations throughout Guangxi. Considering the limited sample size, we could only establish a preliminary prevalence trend for these STs. Future research with a larger sample cohort is necessitated to confirm and further investigate the concurrent dominance of multiple STs in Guangxi Province (37). Analysis of the genetic evolutionary tree confirmed the classification of the 61 tested strains into five genotypes (I, II, III, IV, and V). The results indicate that genotype V first appeared in other regions of China several years ago (35) and is now widely distributed throughout both Guangxi and Guangdong Provinces. In addition, the strong genetic similarity observed between the strains isolated in Guangxi and those in other provinces is likely connected to Guangxi’s prominent role as a major pig-producing region in China. The transport and sale of pigs from Guangxi to other provinces, including Guangdong and Jiangxi, may offer a mechanism for the spread of Mhp. This interprovincial movement of pigs offers a plausible explanation for the observation that the majority of genotype V strains from Guangxi analyzed in this study demonstrate a close genetic relationship with strains identified in Guangdong. In the preceding study, we identified ST128 and ST147 in 5 and 6 different regions across Guangxi, respectively, with both indicating significant prevalence throughout the entire province. Our analysis also indicated that ST199, ST197, and ST203 were detected across multiple regions, indicating clear patterns of epidemic spread in Guangxi. A critical point to emphasize is that these five ST types all originated from genotypes II and V. However, our research demonstrated no significant correlation between the geographical origins of the samples and the ST types of the isolated strains, as we found various ST types present in individual regions, while the same ST types appeared across different geographical locations. These observations align closely with findings documented in the majority of international research papers (29), indicating widespread and extensive dissemination of Mhp across Guangxi Province. Our data also demonstrated that Mhp has maintained its presence in Guangxi over an extended time period, leading to the formation of unique independent clusters. Based on these comprehensive findings, we can draw the logical conclusion that the genetic diversity of Mhp observed in Guangxi acts as one of the key factors contributing to the high infection rates of Mhp observed throughout this geographical area.

## Conclusion

Based on the analysis of 1,362 randomly collected samples from Guangxi, China, our results indicated a high incidence rate of Mhp in the region. The study identified ST147 and ST128 as the primary genotypes, which were detected across six and five regions of Guangxi, respectively. Among the newly identified STs, ST197, ST199, and ST203 demonstrated up as the current trending genotypes in Guangxi. All these ST types fell in cluster II and cluster V, which represented the main genotypes currently spreading throughout Guangxi. This research stands as one of the most thorough analyses into the genotypes and geographical distribution of Mhp in Guangxi’s swine populations. Our findings have significantly advanced our knowledge regarding the incidence rates and molecular typing of Mhp in this region, contributing valuable data to China’s local Mhp epidemiological database while offering a scientific foundation for implementing Mhp control and prevention strategies in Guangxi, China.

## Data Availability

The datasets presented in this study can be found in online repositories. The names of the repository/repositories and accession number(s) can be found below: https://www.ncbi.nlm.nih.gov/genbank/, OP123456–OP123527.

## Footnotes

^1^ https://pubmlst.org

^2^ http://www.comparingpartitions.info/index.php?link=Tool
