# Supplementary figures and images for: Epidemiological investigation of porcine Mycoplasma hyopneumoniae in pig herds in Guangxi, China (2022–2023) and genetic diversity analysis based on multilocus sequence typing
Source: Front Vet Sci. 2025 Aug 13;12:1619301. doi: 10.3389/fvets.2025.1619301 (PMC12380675; doi:10.3389/fvets.2025.1619301)

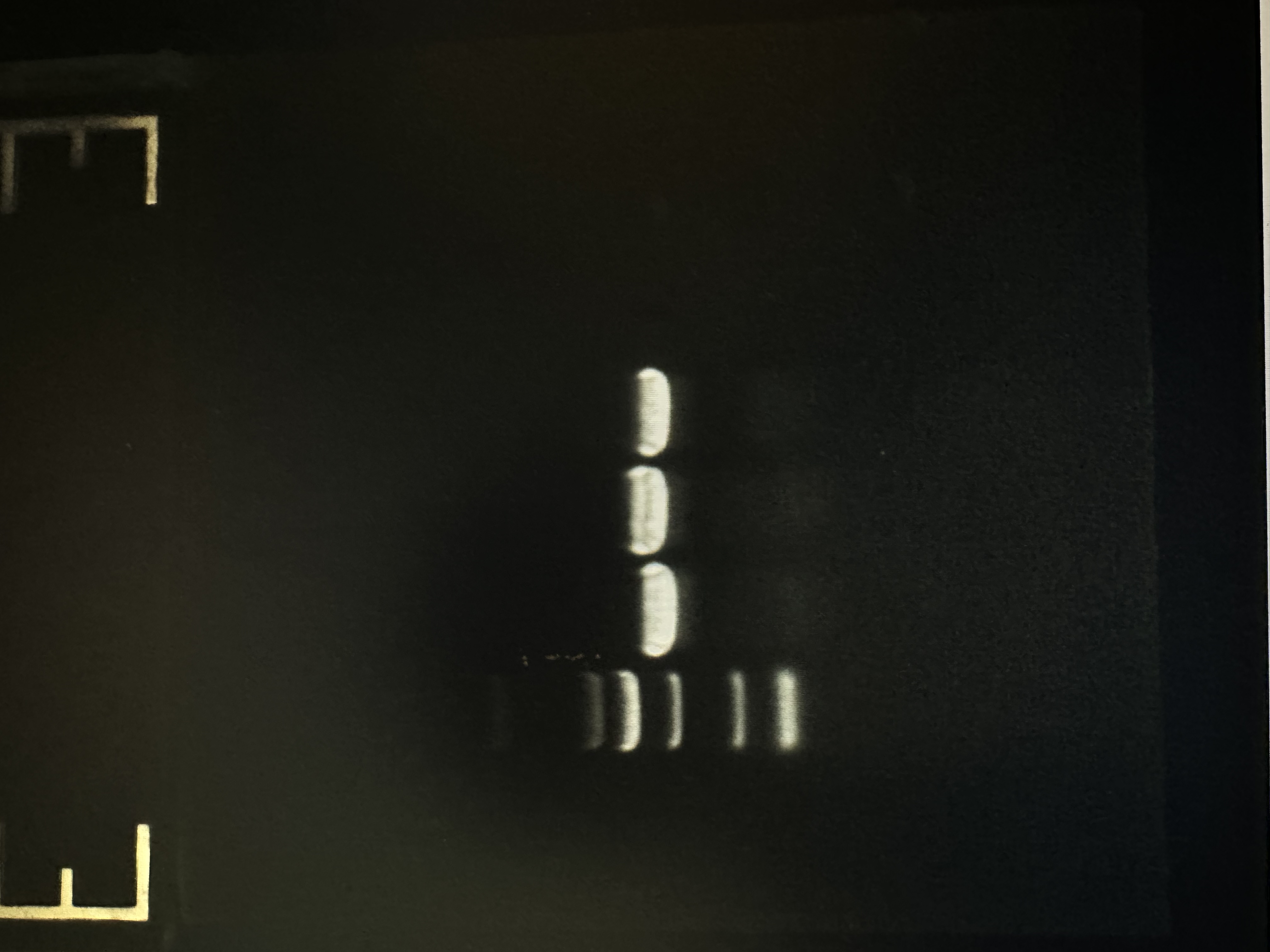

Supplement: Supplementary file 1 [file Image_1.JPEG]
